# Food pantry organizational features, nutrition environments, and partnerships: a community-engaged study in Southern California

**DOI:** 10.1186/s12889-026-26859-7

**Published:** 2026-03-02

**Authors:** Kalani K. Phillips, Ellie Dinh, Isabel F. Almeida, Claudia B. Keller, Denise D. Payán

**Affiliations:** 1 Department of Health, Society and Behavior, Joe C. Wen School of Population & Public Health, University of California, Irvine, USA; 2 Second Harvest Food Bank of Orange County, Irvine, USA; 3 Department of Chicano/Latino Studies and Department of Psychology, School of Social Sciences, University of California, Irvine, USA

**Keywords:** Charitable, Food pantry, Nutrition environment, Partnership, Community-engaged research

## Abstract

**Background:**

Charitable food organizations are critical community hubs for promoting food security and nutrition. Few studies have examined whether food pantry organizational features (e.g., setting and size) are associated with the use of health promotion strategies.

**Methods:**

This cross-sectional study used a validated survey instrument (Nutrition Environment Food Pantry Assessment Tool+, NEFPAT+) in partnership with a southern California regional food bank to examine pantry consumer nutrition environments, including client choice models, marketing, and partnership/referral practices. Eligible pantries were: food bank partners, open at least 1/week for public distribution, and in high-poverty cities. We collected surveys from 27 of 35 eligible sites in 2024. We used descriptive statistics to summarize NEFPAT+ objective outcomes and conducted exploratory analyses to assess differences for specific objective results. Mann‒Whitney U tests and Kruskal‒Wallis tests were used to compare NEFPAT+ scores and objective results by pantry setting and size, respectively.

**Results:**

Surveyed food pantries served a median of 450 households per month (range: 50 − 3,000). 52% were located in faith-based settings and 48% in other community settings. The median NEFPAT+ score was 39 out of 95 points (range: 8–61), indicating most pantries were classified as Silver (37%) or Gold (33%). Overall, 81% of pantries made nutritious foods easy to reach/identify, and 63% used client choice. Only 33% reported using certain marketing strategies. Few used local food sourcing or point-of-decision signage. Most pantries (78%) reported having external partnerships, and 74% referred clients to a local resource. Pantries located in other community settings used nutrition-focused strategies (i.e., client choice, healthy eating signage/recipes) and referred/partnered with community organizations (i.e., food, healthcare, employment assistance) at a significantly higher rate compared to pantries in faith-based settings. We did not find statistically significant differences by pantry size.

**Conclusions:**

This community-engaged study reveals how food pantries may function as multifaceted community health hubs for health promotion interventions. Results show both significant organizational strengths and targeted opportunities for improvement, with strategy use varying by food pantry setting type. Future research should evaluate whether pantries in faith-based settings need additional support, engagement, or technical assistance to promote community health in marginalized communities.

**Supplementary Information:**

The online version contains supplementary material available at 10.1186/s12889-026-26859-7.

## Background

Food insecurity, defined as an economic and social condition associated with decreased access to nutritious foods [1], is a critical public health issue impacting communities across the United States (U.S.) [2, 3]. In 2023, approximately 18 million or 13.5% of U.S. households were food-insecure—an increase from 12.8% in 2022 [4]. Food insecurity is associated with chronic health conditions (e.g., asthma, depression, poor oral health, diabetes [5], and cardiovascular disease [6, 7]), as well as an increased risk of hospitalization [5], a greater risk of premature death, and a shorter life expectancy [8]. Food insecurity also disproportionately impacts certain racial/ethnic groups and low-income communities—people of color (i.e., Latino and Black) and other marginalized communities experience the highest food insecurity levels [6, 9, 10], emphasizing the potential impacts of structural barriers and systemic racism on access to nutritious foods [11].

Community-based organizations (CBOs), including charitable food organizations, play a vital role in responding to these challenges, and can provide direct food assistance and implement innovative strategies to improve food access in underserved communities [12, 13]. Given these serious health consequences and persistent disparities, assessing how CBOs operate and partner can reveal important and feasible strategies to improve community health.

The charitable food system, comprised of food banks and pantries, seeks to alleviate hunger and food insecurity in the U.S [14]. An estimated 49,000 + food pantries and 200 food banks distribute free food [15] and other items (e.g., hygiene, personal care items) to resource-limited households in the U.S. Many households visit food pantries regularly to alleviate chronic food insecurity and rely on pantries as a key food source. In 2020, 36.5% of food-insecure households in the U.S. reported using food pantries [16], and research has indicated that many clients have sustained reliance on food pantries [17]. State-level studies also show similar patterns. A 2017 survey of Minnesota food pantry clients (*n* = 4,321) revealed that 74.4% relied on the same pantry for more than a year, and the majority said that they obtained at least half of their total food from a pantry [18].

Although food pantries serve as crucial access points for households experiencing food insecurity, few studies have examined food pantry consumer nutrition environments. Consumer nutrition environments—the aspects of food settings that influence nutritious food selection—encompass factors such as client choice options (i.e., allowing clients to select specific foods rather than receive pre-packed boxes), marketing and nudging strategies (e.g., strategic food placement, signage, recipes), and information provisions that can shape dietary behaviors [12, 19–22]. Whether a food pantry places fresh produce in a prominent, easy-to-reach area or tucked away in the back can significantly influence client selection, as studies have shown that strategic food placement increases the selection of healthier items [23–25]. Prior research has focused primarily on validating a nutrition ranking system based on the Healthy Eating Research (HER) nutrition guidelines in charitable food organizations [26], and evaluating policy, systems, and environmental interventions [27], with limited investigation of food pantry consumer nutrition environments. Additionally, food pantries and many non-brick-and-mortar food resources are often excluded from local food environment assessments [28].

Unlike many other food store types, food pantries can vary widely in terms of their organizational structure and capacity to provide nutrient-rich foods or meet local demand. They are often located in a variety of settings ranging from faith-based organizations to schools and other secular community spaces, and may rely on volunteers for key personnel with limited operational times per week [15]. Food pantries also face numerous operational challenges, including an inconsistent food supply, insufficient or inadequate food storage, staffing limitations [29], client-facing barriers (i.e., transportation or financial constraints) [30], and linguistic and cultural barriers [31]. The nutritional environment of food pantries and strategies they use to promote health are influenced by these operational constraints as well as policy, food costs, personnel and economic resources, storage capacity, and food donors [12, 19–22, 31, 32]. Understanding how organizational characteristics shape these consumer nutrition environments is critical to develop targeted interventions that increase health equity, as different types of organizations may have varying capacity, resources, and approaches to serve marginalized communities [29, 30, 33].

Despite their central role in addressing food insecurity, research gaps remain on the capacity of food pantries to serve as intervention sites that link clients to preventive health services [34–36], as well as on pantry consumer nutrition environments themselves. Beyond direct food provision, food pantries can serve as community hubs to link clients with local resources and information. Community-based partnerships between clinics and food pantries can improve access to health services and address diet-related issues [34], which has been the basis for co-locating pantries in primary clinical settings [35], or referring patients from food-insecure households to nearby food pantries as part of the Food is Medicine movement.

Few studies have examined how CBOs (e.g., organizations to improve education, healthcare, social welfare, etc.) interact or partner with charitable food programs. Given the complexity and scale of food insecurity in the U.S., coupled with frequent client use of food pantries, assessing partnerships and networks between CBOs and the charitable food system can help inform effective community-based interventions. A systematic review revealed that while food aid interventions alone can help reduce food insecurity levels, programs paired with community support services (e.g., social, health, and welfare resources) are more effective at both decreasing food insecurity and providing additional resources to support health and wellbeing [36].

This article describes the consumer nutrition environments of food pantries in high-poverty cities in a large county in southern California. We particularly focus on the following: (1) client choice models for nutritious food selection, (2) marketing and nudging strategies to promote healthy foods, and (3) partnerships and referral practices that connect clients to additional community resources. We also conduct exploratory analyses to assess whether pantry organizational characteristics such as food pantry size and setting are associated with these strategies and practices.

## Methods

The study uses a quantitative cross-sectional observational survey design to examine the consumer nutrition environment in a sample of urban food pantries.

### Study setting and community-engaged approach

The study setting is Orange County, a large predominantly urban county with high income inequalities and food insecurity disparities located in southern California. In 2023, this county’s food insecurity rate was 11.4%, or approximately 359,910 individuals (population: >3 million people) [37]. In terms of racial/ethnic disparities, 22% of Black and 22% of Latino county residents are estimated to be food insecure, whereas 8% of White residents are food insecure [37].

Due to these community characteristics and disparities, we used a community-engaged approach [38] to design and conduct this study. Our approach was grounded in reciprocity and mutual benefits between academic and community partners. Research questions and the sampling approach were developed through an academic‒community partnership between a large public university and regional food bank. The food bank is an established community organization with high leadership and organizational capacity and an extensive regional partnership network. Their mission statement emphasizes partnering to “provide dignified, equitable and consistent access to nutritious food” as a foundation for community health [39].

Before data collection, the research team shared relevant work with food bank personnel, discussed methodological approaches and strategies, and elicited feedback/answered questions to promote transparency, clear communication, and collaboration. We used a multi-pronged dissemination plan to promote data sharing and evidence-based action. Each participating pantry received a comprehensive individual report detailing their results, score classification, and tailored nutrition environment recommendations with an opportunity to further discuss results with the research team. A sample of an individual pantry report is included (see Additional File 1). The food bank received a general report with cumulative results.

### Theoretical basis

This research draws on organizational capacity and social capital theories to guide exploratory analyses on pantry size and setting. Organizational capacity theory posits that an organization’s ability to achieve its mission depends on its human, financial, and structural resources [40]. Applied to food assistance programs, research shows that capacity attributes such as staffing, infrastructure, and operational resources directly influence organizational effectiveness [40], whereby larger food pantries may have greater operational capacity to adopt health and nutrition promotion strategies like client choice use or to partner with other CBOs [41]. Additionally, social capital theory emphasizes how networks and relationships facilitate collective action [42], therefore the social capital available to a food pantry from its host organization (i.e., setting) may shape or influence their partner referral network. For example, faith-based organizations may leverage more social capital within their congregation while other community organizations may leverage or cultivate external partnerships with other CBOs or government programs [42].

### Recruitment and data collection

The partnering food bank provided a census of partner food distribution sites (as of 11/2023) open to the public, excluding shelters or potentially sensitive locations. From this list, we identified a sample of pantries that met the following criteria: (1) located in the target county; (2) open at least once per week for public distribution; and (3) located in a city with a ≥ 10% poverty rate and ≥ 35% Latino residents. Excluded sites were open < 1 per week, served hot/prepared meals, exclusively delivered meals, or did not meet other eligibility criteria.

A total of *N* = 35 food pantries operating across six cities were eligible. Key operations personnel (i.e., food pantry leadership, liaisons, volunteer leads) from these pantries were emailed by the food bank and invited to complete one online survey per pantry between March and June 2024. Surveys could be completed by the primary pantry contact with assistance and input from other pantry personnel. Incomplete online survey entries (< 25% complete) were deleted, and research staff contacted these sites to provide data collection support. We deployed trained research staff for in-person data collection if the pantry personnel expressed a preference for this option. Four surveys were completed by hand and emailed to us, 8 were collected in-person, and 15 were collected via Qualtrics. Data collected in-person or by hand was input into Qualtrics. Research staff completed a 3-part training, which included a Nutrition Environment Food Pantry Assessment Tool Plus (NEFPAT+) review, pilot data collection session at a university food pantry, and post-data collection debriefing session.

Participating food pantries received a $50 cash incentive. Incentives were distributed to either the food bank’s primary pantry contact or using the contact information provided during data collection. Study protocols and procedures were considered non-human subjects research by self-determination at the University of California, Irvine (#4456).

### Survey tool and measures

We used the NEFPAT + to assess the consumer nutrition environment of food pantries and evaluate a food pantry’s progress in promoting health and nutrition [27, 43, 44]. The NEFPAT + is an updated version of the original NEFPAT survey instrument, which was revised by the tool’s original developers to incorporate updated national guidelines and evidence and include an additional objective assessing partnerships and community resources [43]. The tool has been validated and used in previous research to assess food pantry consumer nutrition environments [43, 44]. The seven objectives are: (1) offers a variety of fruits and vegetables; (2) offers nutritious food from each MyPlate food group; (3) increases client choice for nutritious foods; (4) markets, promotes, and nudges nutritious foods; (5) has guidelines for food safety, nutrition, and customer service; (6) prioritizes health and inclusion in pantry operations; and (7) promotes partnerships and provides additional resources. *Nutritious* is defined as any health-promoting food from one of the five MyPlate food groups: fruits, vegetables, proteins, grains, and dairy [44].

Specific objectives are supported by existing evidence on tools and strategies for nutritious item selection. For example, objective 3 focuses on the client choice model, which allows clients to select specific food items [13, 22, 25, 45]. Client choice is linked to greater availability of nutritious foods [30], reduced food waste [13, 20, 45], improved client satisfaction [13, 45], and, in some studies, better mental health outcomes when combined with support services [36]. Each objective has a binary response and is marked as present or not present at the time of observation. Points are summed for each objective and a total cumulative score. Pantries can be classified into one of five categories based on their total score: Diamond (69–95 points), Platinum (52–68), Gold (35–51), Silver (18–34), or Bronze (0–17).

In addition to standard NEFPAT+ measures, we created a food pantry size proxy using reported data on households served per month. This approach is based on work that examined nutritional quality and food pantry size (i.e., measured as the quantity of food distributed) in *N* = 75 pantries in Baltimore, MD [41]. Our proxy for pantry size used the estimated number of households served per month provided by the pantry staff. Two pantries had missing data and only provided data on the number of individuals served per month. We created a range estimate for households per month by multiplying the number of individuals per month by 2 and 4. Then, for the nine pantries with a numeric range, we used the midpoint to standardize the responses. Based on the rounded tertile distribution of these midpoints, we classified pantries as follows: small (< 500 households served per month), medium (500–1000 households served per month), and large (> 1000 households served per month).

### Pantry setting classification

Pantry settings were classified as being located in either a “faith-based” or “other community setting” contingent on the organizational affiliation and physical location of each food pantry. Faith-based pantries were operated by or housed in religious organizations (e.g., churches or other religious sites). Other community settings included general community spaces, recreational sites, schools/youth centers, local businesses, and non-religious non-profit organizations. This classification was determined using information from the food bank or food pantry personnel and confirmed during data collection and incentive distribution.

### Data management and analysis

Survey data were input into Qualtrics and then cleaned and analyzed via Microsoft Excel and Stata 19 BE. The NEFPAT+ objective and cumulative scores were summed. We calculated frequencies and descriptive statistics for the NEFPAT+ objectives and cumulative scores by pantry size and setting (i.e., faith-based or other community settings). Since data were collected using multiple modes (self-report by pantry staff vs. research staff observation), we also compared NEFPAT+ scores by data collection method using a Mann-Whitney U test to assess potential mode effects.

We conducted exploratory analyses to assess differences in specific NEFPAT+ objective results (Objectives 3, 4, and 7) by food pantry size and setting. These objectives were selected because they directly aligned with our stated research focus on client choice, marketing strategies, and community partnerships. For Objective 7, we created a binary (yes/no) composite measure to examine partnership and referral patterns. The binary measure was defined as providing or referring clients to at least one of the following services: external partnerships (i.e., healthcare/public health organizations, cultural groups, universities/schools, youth groups, or community coalitions), educational programming (i.e., nutrition/cooking, gardening, finances, or health-related programs), referral services (i.e., the Supplemental Nutrition Assistance Program (SNAP), other food assistance, healthcare, mental health, employment, self-improvement, physical activity, or services for specific populations), and transportation/mobility services (i.e., mobile markets, home delivery, or transportation vouchers).

We also used Kruskal‒Wallis tests to examine differences in the NEFPAT+ scores by pantry size category (small, medium, and large). Mann‒Whitney U tests were used to compare overall scores and specific NEFPAT+ objective results by setting (faith-based vs. other community settings). Chi-square or Fisher’s exact tests were used to assess associations between pantry characteristics and specific objective results, including binary composite scores. Statistical significance was determined at *p* ≤ 0.05.

## Results

Among 35 eligible food pantries, we collected complete surveys for *N* = 27 pantries (77.1% response rate). Nineteen (70%) questionnaires were self-reported by the pantry staff, and 8 (30%) were completed by trained research staff in-person. No statistically significant differences in NEFPAT+ scores were observed by collection mode.

Table 1 provides information about the organization and setting characteristics of the participating food pantries (*N* = 27), which were located across five cities. Most pantries (71%; *n* = 19) were in large cities with a population of 300,000 + residents, and only 2 (7%) were in small cities with < 101,000 residents. Cities had poverty rates ranging from 10 to 13% [46].

**Table 1:** Food pantry organization and setting characteristics (*N* = 27) by size and setting

Characteristics	Total *N* = 27	Food Pantry Size			Food Pantry Setting	
		Small (< 500 households)	Medium (500–1000 households)	Large (> 1000 households)	Faith-based	Other community setting^1^
Organization type, *n* (%)	27 (100%)	14 (52%)	6 (22%)	7 (26%)	14 (52%)	13 (48%)
City size, *n* (%)						
Large city (300,000+)	19 (71%)	11 (79%)	2 (33%)	6 (86%)	10 (71%)	9 (70%)
Medium city (101,000-200,000)	6 (22%)	2 (14%)	3 (50%)	1 (14%)	4 (29%)	2 (15%)
Small city (< 101,000)	2 (7%)	1 (7%)	1 (17%)	0 (0%)	0 (0%)	2 (15%)
Households served/month, median (range)	450 (50–3,000)	238 (50–450)	658 (545–900)	1,726 (1,200-3,000)	350 (50 − 3,000)	545 (120-1,800)
Individuals served/month, median (range)^2^	924 (45–11,000)	737 (45–1,500)	1,525 (100–2,500)	5,869 (400–11,000)	659 (45–11,000)	1,300 (200–6,738)

^1^Other community-based settings include: general community spaces, recreational sites, schools/youth centers, businesses, and non-profit organizations

^2^Three pantries had missing data for individuals served/month and are not reported

Surveyed food pantries varied in size and setting. Over half (52%; *n* = 14) were categorized as small, serving < 500 households per month. 22% were categorized as medium, serving 500–1,000 households per month, and 26% (*n* = 7) were large pantries serving > 1,000 households per month. The median number of households served per month was 450 (range: 50 − 3,000), and the median number of individuals served per month was 924 (range: 45 − 11,000).

Just over half of the pantries were located in faith-based settings (52%; *n* = 14), whereas 48% (*n* = 13) were located in other community settings. The latter included general community spaces, recreational sites, schools/youth centers, businesses, and non-profit organizations.

### NEFPAT+ scores and classifications

Table 2 reports the NEFPAT+ cumulative scores, classification, and objective results across all 7 objectives. The median NEFPAT+ score was 39 (range: 8–61). The surveyed pantries spanned all five NEFPAT+ categories—ten classified as Silver (37%), 9 Gold (33%), 6 Platinum (22%), and 2 Bronze (7%), with no pantry classified as ‘Diamond.’

**Table 2:** Nutrition Environment Food Pantry Assessment Tool (NEFPAT+) score results (*N* = 27) by size and setting

NEFPAT+ Result		Food Pantry Size			Food Pantry Setting	
		Small (< 500 households)	Medium (500–1000 households)	Large (> 1000 households)	Faith-based setting	Other community setting
NEFPAT+ score, median (range)	39 (8–61)	37.5 (8–61)	34 (29–48)	44 (25–54)	32.5 (8–61)	44 (29–55)
NEFPAT+ classification, *n* (%)						
Platinum (52–68)	6 (22%)	4 (29%)	0 (0%)	2 (29%)	3 (22%)	3 (23%)
Gold (35–51)	9 (33%)	4 (29%)	2 (33%)	3 (42%)	2 (14%)	7 (54%)
Silver (18–34)	10 (37%)	4 (29%)	4 (67%)	2 (29%)	7 (50%)	3 (23%)
Bronze (0–17)	2 (7%)	2 (14%)	0 (0%)	0 (0%)	2 (14%)	0 (0%)
NEFPAT+ objective score, median score (range)						
1. Offer a variety of fruits and vegetables (3 pts)	1 (0–3)	1 (0–3)	2.5 (0–3)	3 (0–3)	1 (0–3)	1 (0–3)
2. Offer nutritious foods from each MyPlate food group (24 pts)	12 (1–20)	11 (1–20)	13.5 (6–16)	12 (6–18)	12 (1–20)	12 (6–20)
3. Increase the client’s choice of nutritious foods (6 pts)	4 (0–6)	3 (0–6)	4 (2–5)	3 (0–6)	3 (0–6)	4 (1–6)
4. Market, promote, and nudge nutritious foods (6 pts)	2 (0–5)	3 (0–5)	2 (0–3)	2 (0–5)	2 (0–5)	3 (1–4)
5. Has guidelines for food safety, nutrition, and customer service (12 pts)	4 (1–12)	5 (1–10)	2.5 (2–9)	5 (2–12)	3 (1–10)	7 (2–12)
6. Prioritizes health, and inclusion in pantry operations (14 pts)	7 (0–12)	6.5 (0–12)	8 (3–8)	6 (0–12)	5.5 (0–12)	7 (3–10)
7. Promotes partnerships and provides additional resources (20 pts)	7 (0–17)	7 (0–17)	4.5 (2–15)	11 (0–14)	3.5 (0–14)	11 (4–17)

No statistically significant differences were identified by pantry size or setting. The Kruskal-Wallis test was used for pantry size comparisons. The Mann-Whitney U test for setting comparisons. Fisher's exact test for score classification distributions

When examining objective scores as a percentage of maximum points, pantries scored highest on objective 3 which assessed strategies to increase the client’s choice of nutritious foods (median: 4/6, 67%; range 0–6). The next highest scores were for objective 2 to offer nutritious foods from each MyPlate food group (median: 12/24, 50%; range: 1–20), and objective 6 to prioritize health and inclusion practices (median: 7/14, 50%; range: 0–12). Pantries scored lowest on objective 1, offering diverse fruits and vegetables (median: 1/3, 33%; range: 0–3); objective 5, guidelines for food safety, nutrition, and customer service (median: 4/12, 33%; range: 1–12); and objective 4, marketing and nudging nutritious foods (median: 2/6, 33%; range: 0–5). No statistically significant differences were found for the NEFPAT+ score results by pantry size or setting.

Next, we explored whether food pantry size or setting was associated with specific NEFPAT+ objective results. Table 3 includes objective 3 (client choice), 4 (marketing and nudging strategies), and 7 (partnerships and referrals) results, and examines differences by pantry setting. The food pantry size results are not included in this table since no statistically significant results were found for this organizational characteristic (see Additional File 2).

**Table 3:** NEFPAT+ results for client choice, marketing strategies, community partnerships/information (*N* = 27) by setting

Pantry Practice	Total *N* = 27	Food Pantry Setting		
		Faith-based *N* = 14 (52%)	Other community setting *N* = 13 (48%)	*p*-value
Objective 3. Client choice for nutritious foods				
Clients can shop more than once per month	23 (85%)	12 (86%)	11 (85%)	1.00
Offers items from each of the 5 MyPlate food groups	22 (81%)	11 (79%)	11 (85%)	1.000
Clients can choose which foods they want from at least one MyPlate food group	17 (63%)	*5 (36%)*	*12 (92%)*	*0.004*
Choice items include *Nutritious* foods	15 (56%)	8 (57%)	7 (54%)	1.000
Uses funds to buy *Nutritious* foods or encourages donations of *Nutritious* foods	13 (48%)	8 (57%)	5 (38%)	0.257
Seeks fresh product from local sources via gleaning, community gardens, having an onsite garden, farms, or other approaches	4 (15%)	2 (14%)	2 (15%)	0.596
Objective 4. Marketing and nudging strategies				
*Nutritious* foods are easy to see and reach or are easy for clients to identify to volunteers	22 (81%)	11 (79%)	11 (85%)	1.000
*Nutritious* foods are displayed before, in front of, or above other foods	12 (44%)	5 (36%)	7 (54%)	0.449
At least one *Nutritious* food is included in a bundle to display items together as a meal or recipe	11 (41%)	7 (50%)	4 (31%)	0.440
Offers recipes to promote *Nutritious* foods to clients	9 (33%)	*2 (14%)*	*7 (54%)*	*0.046*
Uses signage promoting general healthy eating	9 (33%)	*2 (14%)*	*7 (54%)*	*0.046*
Uses point-of-decision signage to encourage selection of specific items	3 (11%)	1 (7%)	2 (15%)	0.596
Objective 7. External partnerships, programming, referrals, and services				
At least one partnership	21 (78%)	9 (64%)	12 (92%)	0.165
Community coalition or network	16 (59%)	7 (50%)	9 (69%)	0.440
Healthcare or public health organization	15 (56%)	5 (36%)	10 (77%)	0.054
University, college, or school	11 (41%)	4 (29%)	7 (54%)	0.252
Culture/ population specific group	7 (26%)	2 (14%)	5 (38%)	0.209
Youth group or 4-H club	7 (26%)	3 (21%)	4 (31%)	0.678
Provides at least one educational component/program	17 (63%)	*6 (43%)*	*11 (85%)*	*0.046*
Health related programs or screening	13 (48%)	4 (29%)	9 (69%)	0.057
Nutrition or cooking	10 (37%)	3 (21%)	7 (54%)	0.120
Finances	9 (33%)	*1 (7%)*	*8 (62%)*	*0.004*
Gardening	0 (0%)	0 (0%)	0 (0%)	---
Provides at least one referral service	20 (74%)	8 (57%)	12 (92%)	0.077
Supplemental Nutrition Assistance Program (SNAP) application support	17 (63%)	*6 (43%)*	*11 (85%)*	*0.046*
Other food assistance programs	17 (63%)	*6 (43%)*	*11 (85%)*	*0.046*
Medicaid and/or affordable healthcare	14 (52%)	*4 (29%)*	*10 (77%)*	*0.021*
Mental health services	12 (44%)	*2 (14%)*	*10 (77%)*	*0.002*
Services to meet specific population needs	12 (44%)	*3 (21%)*	*9 (69%)*	*0.021*
Self or family-improvement	11 (41%)	3 (21%)	8 (62%)	0.054
Employment	9 (33%)	*2 (14%)*	*7 (54%)*	*0.046*
Physical activity	3 (11%)	0 (0%)	3 (23%)	0.098
Provides at least one transportation or mobility service	12 (44%)	5 (36%)	7 (54%)	0.464
Home delivery	11 (41%)	5 (36%)	6 (46%)	0.704
Transportations vouchers or passes	5 (19%)	2 (14%)	3 (23%)	0.648
Mobile markets	3 (11%)	1 (7%)	2 (15%)	0.596
Overall partnership and referral performance score, median points (IQR)	7 (3–13)	3.5 (0–6)	11 (7–13)	0.064

*Italics* indicate statistically significant findings at *p*≤0.05. Chi-square or Fisher's exact tests used for individual practices and “at least one” practice composite scores due to small cell sizes. Overall partnership and referral performance score uses summed NEFPAT+ Objective 7 scores analyzed with Mann-Whitney U tests; values shown as median (IQR). Binary coding used for each pantry practice. Composite scores indicate pantries providing “at least one” service per category

#### Strategies to increase the client’s choice of nutritious foods (objective 3)

Client choice implementation was common but varied by setting. Many surveyed pantries allowed clients to shop more than once per month (85%; *n* = 23) and offered items from all five MyPlate food groups (81%; *n* = 22). The majority used a client choice model (63%; *n* = 17), and these items included nutritious foods (56%, *n* = 15). Compared with faith-based pantries, client choice was used significantly more in pantries in other community settings (36% vs. 92%; *p* = 0.004).

Nearly half (48%; *n* = 13) used funds to buy nutritious foods or encouraged nutritious food donations. Local food sourcing was a low-use strategy, reported by just 15% (*n* = 4) of pantries who said that they obtained fresh produce from local sources (e.g., gleaning, community or onsite gardens, farms).

#### Strategies to market, promote, and nudge nutritious foods (objective 4)

Most pantries (81%; *n* = 22) made nutritious foods easy to see/reach or easy to identify. Under half displayed nutritious foods paired with another food (44%; *n* = 12) and included at least one nutritious food in a bundle to display a meal or recipe (41%; *n* = 11). A third used signage to promote general healthy eating (33%; *n* = 9) and offered recipes to promote nutritious foods (33%; *n* = 9). Few pantries used point-of-decision signage to encourage item selection (11%; *n* = 3).

The use of recipes or signage to promote nutritious foods/healthy eating was significantly more prevalent among food pantries in other community settings than among pantries in faith-based settings (54% vs. 14%; *p* = 0.046).

#### Strategies to promote partnerships and referrals (objective 7)

Partnership and referral practices were widespread but differed by setting. Overall, 78% (*n* = 21) of pantries reported at least one partnership with external groups. Most partnered with community coalitions or networks (59%; *n* = 16) or healthcare or public health organizations (56%; *n* = 15). Fewer than half reported partnering with a university, college, or school (41%; *n* = 11); and approximately a quarter partnered with either a culture/population-specific group (26%; *n* = 7) or a youth group (26%; *n* = 7). Many pantries in other community-based settings (77%) reported partnering with a healthcare or public health organization compared to just 36% of faith-based institutions—a difference approaching statistical significance at *p* = 0.054.

63% (*n* = 17) of the pantries offered at least one educational component or program. The most frequently offered programs were health-related (48%; *n* = 13) or nutrition/cooking education (37%; *n* = 10). A third offered financial education programming (33%; *n* = 9), and none offered gardening. Food pantries in other community settings were significantly more likely than those in faith-based settings to provide educational programming overall (85% vs. 43%; *p* = 0.046) and financial education (62% vs. 7%; *p* = 0.004).

A high percentage of pantries referred clients to at least one type of local resource (74%; *n* = 20). Referrals were defined as providing clients with information about available services, assisting clients with applications or enrollment, or directly connecting clients with partnering organizations. Most commonly, they provided SNAP application support (63%; *n* = 17) or referred clients to other food assistance programs (63%; *n* = 17), which was significantly more common in pantries in other community settings (*p* = 0.046 for both). More than half of the pantries reported referring clients to Medicaid/affordable healthcare (52%; *n* = 14); fewer than half reported referring clients to mental health services (44%; *n* = 12) and services for specific population needs (44%; *n* = 12). Pantries in other community settings were significantly more likely to provide Medicaid/healthcare referrals (77% vs. 29%; *p* = 0.021), mental health referrals (77% vs. 14%; *p* = 0.002), and referrals for specific population needs (69% vs. 21%; *p* = 0.021) compared to those in faith-based settings. Few pantries offered employment (33%; *n* = 9) or physical activity resource referrals (11%; *n* = 3) – both were significantly more common in other community settings than in faith-based settings.

44% (*n* = 12) of the pantries provided at least one mobility service to address client transportation barriers. For individual practices, 41% offered home delivery (*n* = 11), 19% offered transportation vouchers/passes (*n* = 5), and 11% had mobile markets (*n* = 3).

Overall partnership and referral performance varied by pantry setting. Other community settings had higher median partnership and referral scores (median: 11; interquartile range [IQR]: 7–13), compared to faith-based settings (median: 3.5; IQR: 0–6). However, this difference was not statistically significant (*p* = 0.064).

## Discussion

Importantly, this study examines key components of food pantry nutrition environments and strategies/practices used to promote nutrition. Results reveal that while some health promotion strategies may be widely used to promote healthy eating, others are underutilized. In our sample of *N* = 27 food pantries in high-poverty cities in a large southern California county, 81% offered items from all MyPlate food groups and made nutritious foods easy to access/identify. However, marketing strategy implementation was limited, with only 33% using signage or recipes to promote client selection of nutritious foods, and 15% using local food sourcing strategies. Evidence-based marketing strategies such as nutrition signage, recipe provision, and strategic food placement are relatively low-cost approaches that can improve these facets without restricting client choice [18, 23, 47–49]. Technical assistance interventions, which provide professional expertise to support organizational improvements, have shown significant increases in both the marketing strategies used and the overall NEFPAT+ scores following implementation [50]. When combined with evidence-based marketing strategies, these approaches can improve nutrition environments without restricting individual preferences, offering cost-effective and culturally adaptable approaches for pantries to promote wellness while respecting client choice [18, 49].

Across all objectives, strategies with lower adoption rates, including point-of-decision signage (11%), local food sourcing (15%), and signage or recipes promoting nutritious foods (33%), represent opportunities for targeted support or technical assistance. However, not all pantries implemented strategies equally. The study assessed associations between organizational characteristics and the availability of specific strategies and practices, finding significant differences by setting type. Compared with pantries in faith-based settings, food pantries located in other community settings more commonly implemented nutrition-focused strategies and partnership activities, including client choice models and healthy eating signage and recipes. These differences are particularly important given the potential of nudging strategies to improve client outcomes. Nudging strategies have been shown to increase the selection of healthy foods and healthier eating habits among clients, although evidence of long-term changes in diet or health is limited [22, 23, 47, 48]. The client choice model may also increase the likelihood that clients select foods matching their cultural and dietary preferences [13, 20, 33, 45]. In our sample, only 63% of the pantries used this model, with higher implementation in other community setting pantries. This finding is consistent with prior literature and reveals that setting type may be a more influential organizational feature than pantry size alone [41, 51]. Specifically, a recent study of rural food pantries found organizational differences in service delivery approaches [51], reflecting the unique organizational contexts, resources, and constraints that different types of community organizations, such as food pantries, face.

In terms of referrals and community partnerships, a high percentage (78%) of the sampled food pantries reported existing partnerships with local CBOs. Many pantries also reported that they already partnered with healthcare organizations (56%) and provided referral services. These are promising avenues to leverage for future community health interventions. A systematic review of food pantry interventions in the U.S. revealed that while food assistance alone can be helpful, involving nutrition education and/or collaboration with health professionals is associated with more successful and sustainable interventions, suggesting that integrated community support services may be more effective in addressing the complex needs of food-insecure households [52].

This pattern of organizational differences also extends to community connections and referrals. Food pantries located in other community settings implemented certain nutrition strategies and connected clients to external resources at higher rates than faith-based pantries did. Differences in referral rates were particularly notable for nutrition assistance programs (85% vs. 43%) and employment support (54% vs. 14%). Future research should continue to examine these associations by setting type and evaluate whether pantries in faith-based settings need additional support, engagement, or technical assistance for improvement. Studies with larger and more geographically diverse samples could also test whether these exploratory findings are generalizable, and identify mechanisms underlying these organizational differences.

The findings of this study reveal specific opportunities to improve pantry consumer nutrition environments and the development of multi-level interventions to improve food security, diet, and health. Evidence suggests that food pantries can serve as effective intervention sites to connect clients to preventive health services when properly supported [27, 34–36], with technical assistance programs showing significant improvements in consumer nutrition environments measured by the NEFPAT+ [50]. For example, federal nutrition programs such as SNAP play a critical and complementary role in charitable food interventions. Programs such as SNAP have been proven to reduce food insecurity and decrease reliance on emergency food systems [5, 53]. Our finding that 63% of pantries provided SNAP application support underscores their capacity to serve as effective referral units [54]. When such supports are reduced or unavailable, food pantries often serve as a final safety net [55, 56]. Initiatives that integrate SNAP benefits, Medicaid, or referrals to healthcare providers or interventions like the Live Well Alabama Healthy Food Pantry—which relied on partnered technical assistance and support from a government food assistance education program [57]—may be important for effectively improving health outcomes and supporting the charitable food system.

Currently, there is substantial interest and investment among health care delivery organizations to partner with nonmedical organizations in addressing complex health and social needs for patients with diet-related chronic illnesses, such as type 2 diabetes [58]. However, most of this work focuses on partnering with existing food distribution sites rather than strengthening or improving the nutrition environment of food distribution sites and retailers in marginalized communities. Policymakers and practitioners should consider supporting bidirectional information networks between public agencies and the charitable food system to streamline referrals [59, 60]. In the context of federal threats to nutrition assistance programs, strengthening local charitable food systems is essential. Although sites often need additional training and/or technical assistance, food pantries have the potential to provide referrals effectively [54].

When the charitable food system collaborates with or receives investments from federal resources and healthcare services, programming becomes more sustainable [59, 60], health outcomes improve, and food insecurity is reduced [61, 62]. Given the ongoing federal policy uncertainties around food aid programs such as SNAP [63] and healthcare coverage, it is increasingly important for communities to strengthen local food assistance networks and partnerships.

### Strengths and limitations

Study limitations include a small sample size (*N* = 27) based in southern California, which may have limited generalizability and statistical power for subgroup analyses, particularly for pantry size. Future research conducted with a larger sample and in different geographic regions should further investigate whether pantry size is associated with healthy eating strategies and nutritious food availability. Next, the cross-sectional design does not allow for causal inferences, and self-reported data may be subject to bias. Data were collected using multiple modes which may have led to a higher response rate; however, it could have led to potential bias effects (e.g., social desirability or recall bias for self-reported surveys). Although no statistically significant differences were found by collection method (*p* = 0.958), small subsample sizes limit our ability to assess mode effects for descriptive or exploratory analyses. Finally, the NEFPAT+ does not capture client satisfaction, food cultural relevance, or health outcomes. Food access challenges can intersect with cultural preferences, which underscores the importance of culturally relevant food offerings [64] and practices in consumer nutrition environments. While the NEFPAT+ assesses the availability of some inclusive practices (e.g., translated materials), it does not evaluate whether the food offered is aligned with the cultural preferences and dietary needs of the communities being served. Future research might focus on developing survey measures based on client experience data to assess these important elements, like cultural inclusion, preferences, or connection with food [31, 65]. Additionally, while this study focused on a quantitative assessment of food pantry consumer environments, future research might use mixed or qualitative methods to explore contextual factors underlying any differences by setting type or to capture client perspectives on pantry services.

Study strengths include a high response rate from a targeted food pantry sample, the use of a validated consumer nutrition environment tool, and the use of a community-engaged approach. Partnering with an established local CBO with a strong network and trust can be a powerful vehicle for improving data collection efforts and addressing upstream factors that are key social determinants of health, including food insecurity. This approach takes unique community factors into consideration and fosters collaboration, learning, and capacity building to promote health equity [38, 66]. Providing reports to participating pantries was prioritized, which is an important step to support local data-informed decisions among community participants and organizations.

## Conclusions

Charitable food organizations are an essential part of the nutrition assistance landscape, particularly in underserved communities. This community-engaged study reveals how food pantries can function as multifaceted community health hubs for targeted, evidence-based interventions. The results reveal both significant organizational strengths and targeted opportunities for improvement, with strategy use varying by food pantry setting type.

## Supplementary Information

Additional File 1: Sample Food Pantry Assessment Report. PDF document (Additional File_1.pdf) showing an example of the individualized feedback reports we provided to participating food pantries, including their NEFPAT+ scores, classification, and tailored recommendations based on the nutrition environment assessment we completed.

Additional File 2: Table S1. NEFPAT+ results for client choice, marketing strategies, and community partnerships by pantry size (*N*=27). Word document (Additional File_2.docx) showing client choice, marketing strategies, and community partnership results for small (<500 households), medium (500-1000 households), and large (>1000 households) food pantries (*N*=27).

## Data Availability

Select aggregate data may be shared upon reasonable request to the senior author (DDP). Full data are not available since this request was not communicated to personnel when study information was provided.

## Abbreviations

NEFPAT+: Nutrition Environment Food Pantry Assessment Tool Plus
U.S.: United States
CBOs: Community-based organizations
SNAP: Supplemental Nutrition Assistance Program
HER: Healthy Eating Research
IQR: Interquartile range
