# Supplementary material for: Food pantry organizational features, nutrition environments, and partnerships: a community-engaged study in Southern California
Source: BMC Public Health. 2026 Mar 2;26:1132. doi: 10.1186/s12889-026-26859-7 (PMC13059207; doi:10.1186/s12889-026-26859-7)

Food Pantry Assessment

**Food Pantry Site**	[Food Pantry Name]
**Assessment Date**	[Date]

Background & Purpose

The following is an assessment of the consumer nutrition environment at **[Food Pantry Name]** using the validated Nutrition Environment Food Pantry Assessment Tool (NEFPAT). The NEFPAT is a nutrition environmental assessment tool to help identify food pantry strengths and strategies to help promote healthier choices among clients. NEFPAT data on your pantry was collected as part of a larger research study led by UCI Public Health faculty Dr. Denise Payán in partnership with Second Harvest Food Bank of Orange County. This report includes the following sections:

- Background & Purpose
- Methods
- NEFPAT Score & Pantry Rating
- NEFPAT Score Results by Objective

Methods

The NEFPAT is a nutrition environmental assessment tool with 7 objectives (listed on the next page) to track food pantry strengths and to identify strategies to help promote healthier choices among clients.

The NEFPAT defines *Nutritious* as any health-promoting foods, using MyPlate as a nutrition guide. MyPlate has five food groups, including fruits, vegetables, proteins, grains, and dairy. MyPlate also helps individuals on a budget select and plan meals that promote healthy eating.

NEFPAT Score & Pantry Rating

0-17	18-34	35-51	52-68	69-85
Bronze	Silver	Gold	Platinum	Diamond

36 Points

NEFPAT Score Results by Objective

Below is a summary report listing strengths and recommendations that can help enhance the nutrition environment of your pantry for clients. If you are interested in any resources for a specific recommendation below, please let our team know **[EMAIL]**. We can share resources developed by the University of Illinois Extension program.

Strength	Highlighted in Green
Approaching Strength	Highlighted in Yellow
Area for Improvement	Highlighted in Orange

Objective & Description	Score
**1. Offers a Variety of Fruits and Vegetables:**<br>**Strength/s:** At least 5 different types of fruits are available<br>**Recommendation/s:** To provide at least 5 different types of vegetables	1/3
**2. Offers Nutritious Foods from each MyPlate Food Group**<br>**Strength/s:** Offers a wide variety of fruit, vegetable, and grain options<br>**Recommendation/s:** To offer additional dairy options such as low-fat yogurt and cottage cheese	11/24
**3. Increase Client Choice for Nutritious Foods**<br>**Strength/s:** Offers items from each of the 5 MyPlate food groups to clients more than once a month<br>**Recommendation/s:** To consider seeking fresh produce from sources such as community gardens	3/6
**4. Market, Promote, and Nudge Nutritious Foods**<br>**Strength/s**: Nutritious foods are displayed and easy to reach for clients<br>**Recommendation/s:** To use signage to encourage selection of Nutritious foods and healthy eating	4/6
**5. Has Guidelines for Food Safety, Nutrition, and Customer Service**<br>**Strength/s:** Ensures safe food handling and has a nutrition policy that is reviewed at least once a year<br>**Recommendation/s:** To consider using digital platforms to market services	7/12
**6. Prioritizes Health & Inclusion in Pantry Operations**<br>**Strength/s:** Has a system to identify the nutritional value of foods and provides volunteer education<br>**Recommendation/s:** To ask about client needs such as culturally familiar food and dietary needs	5/14
**7. Promoted Partnerships & Provides Additional Resources**<br>**Strength/s:** Provides information about local resources including food assistance and Medicaid<br>**Recommendation/s:** To provide information on additional resources such as employment and mental health services	5/20

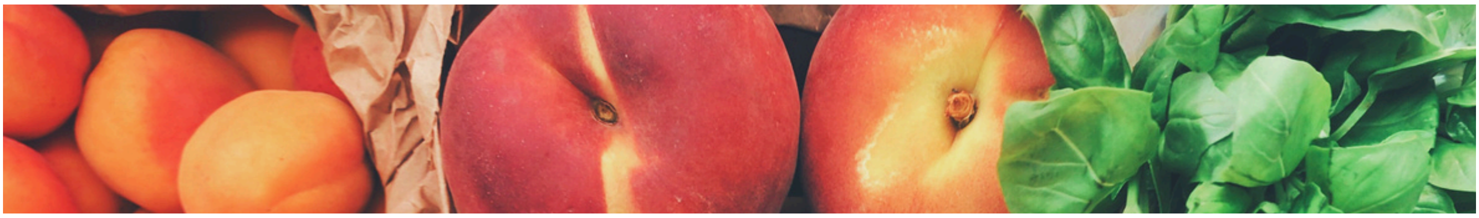

Supplement: Supplementary file 1 — Additional File 1: Sample Food Pantry Assessment Report. PDF document (Additional File_1.pdf) showing an example of the individualized feedback reports we provided to participating food pantries, including their NEFPAT+ scores, classification, and tailored recommendations based on the nutrition environment assessment we completed. [file 12889_2026_26859_MOESM1_ESM.pdf]
