# Supplementary material for: Food pantry organizational features, nutrition environments, and partnerships: a community-engaged study in Southern California
Source: BMC Public Health. 2026 Mar 2;26:1132. doi: 10.1186/s12889-026-26859-7 (PMC13059207; doi:10.1186/s12889-026-26859-7)
Supplement: Supplementary file 2 — Additional File 2: Table S1. NEFPAT+ results for client choice, marketing strategies, and community partnerships by pantry size (N=27). Word document (Additional File_2.docx) showing client choice, marketing strategies, and community partnership results for small (<500 households), medium (500-1000 households), and large (>1000 households) food pantries (N=27). [file 12889_2026_26859_MOESM2_ESM.docx]

**Table S1.** NEFPAT+ results for client choice, marketing strategies, and community partnerships (N-27) by pantry size. *{No statistically significant differences were found by food pantry size for any measures below)*

| **Pantry Practice** | **Total**  **N=27** | **Food Pantry Size** | | | |
| --- | --- | --- | --- | --- | --- |
|  |  | Small (<500 households) N=14 (52%) | Medium (500-1000 households) N=6 (22%) | Large (>1000 households) N=7 (26%) | p-value |
| **Objective 3. Client Choice for Nutrition Foods** | | | | | |
| Clients can shop more than once per month | 23 (85%) | 11 (79%) | 6 (100%) | 6 (86%) | 0.254 |
| Offers items from each of the 5 MyPlate food groups | 22 (81%) | 12 (86%) | 4 (67%) | 6 (86%) | 0.571 |
| Clients can choose which foods they want from at least one MyPlate food group | 17 (63%) | 6 (43%) | 5 (83%) | 6 (86%) | 0.800 |
| Choice items include *Nutritious* foods | 15 (56%) | 5 (36%) | 4 (67%) | 6 (86%) | 0.699 |
| Uses funds to buy *Nutritious* foods or encourages donations of *Nutritious* foods | 13 (48%) | 8 (57%) | 4 (67%) | 1 (14%) | 0.069 |
| Seeks fresh product from local sources via gleaning, community gardens, having an onsite garden, farms, or other approaches | 4 (15%) | 3 (21%) | 0 (0%) | 1 (14%) | 0.617 |
| **Objective 4. Marketing and Nudging Strategies** | | | | | |
| *Nutritious* foods are easy to see and reach or are easy for clients to identify to volunteers | 22 (81%) | 12 (86%) | 4 (67%) | 6 (86%) | 0.571 |
| *Nutritious* foods are displayed before, in front of, or above other foods | 12 (44%) | 7 (50%) | 2 (33%) | 3 (43%) | 0.786 |
| At least one *Nutritious* food is included in a bundle to display items together as a meal or recipe | 11 (41%) | 7 (50%) | 2 (33%) | 2 (29%) | 0.588 |
| Offers recipes to promote *Nutritious* foods to clients | 9 (33%) | 4 (29%) | 3 (50%) | 2 (29%) | 0.617 |
| Uses signage promoting general healthy eating | 9 (33%) | 5 (36%) | 2 (33%) | 2 (29%) | 0.948 |
| Uses point-of-decision signage to encourage selection of specific items | 3 (11%) | 1 (7%) | 0 (0%) | 2 (29%) | 0.209 |
| **Objective 7. External partnerships, programming, referrals, and services** | | | | | |
| At least one partnership | 21 (78%) | 10 (71%) | 5 (83%) | 6 (86%) | 0.845 |
| Community coalitions or networks | 16 (59%) | 7 (50%) | 4 (67%) | 5 (71%) | 0.675 |
| Healthcare or public health organizations | 15 (56%) | 8 (57%) | 1 (17%) | 6 (86%) | 0.064 |
| Universities, colleges, or schools | 11 (41%) | 6 (43%) | 1 (17%) | 4 (57%) | 0.402 |
| Culture or population specific groups | 7 (26%) | 4 (29%) | 1 (17%) | 2 (29%) | 1.000 |
| Youth groups or 4-H clubs | 7 (26%) | 5 (36%) | 1 (17%) | 1 (14%) | 0.610 |
| Provides at least one educational component/program | 17 (63%) | 8 (57%) | 4 (67%) | 5 (71%) | 0.875 |
| Health related programs or screening | 13 (48%) | 5 (36%) | 4 (67%) | 4 (57%) | 0.412 |
| Nutrition or cooking | 10 (37%) | 5 (36%) | 2 (33%) | 3 (43%) | 1.000 |
| Finances | 9 (33%) | 6 (43%) | 2 (33%) | 1 (14%) | 0.493 |
| Gardening | 0 (0%) | 0 (0%) | 0 (0%) | 0 (0%) | --- |
| Provides at least one referral | 20 (74%) | 10 (71%) | 5 (83%) | 5 (71%) | 1.000 |
| Supplemental Nutrition Assistance Program (SNAP) application support | 17 (63%) | 9 (64%) | 3 (50%) | 5 (71%) | 0.763 |
| Other food assistance programs | 17 (63%) | 9 (64%) | 3 (50%) | 5 (71%) | 0.763 |
| Medicaid and/or affordable healthcare | 14 (52%) | 6 (43%) | 3 (50%) | 5 (71%) | 0.053 |
| Mental health services | 12 (44%) | 5 (36%) | 2 (33%) | 5 (71%) | 0.125 |
| Services to meet needs of specific populations | 12 (44%) | 6 (43%) | 3 (50%) | 3 (43%) | 1.000 |
| Self or family-improvement | 11 (41%) | 7 (50%) | 1 (17%) | 3 (43%) | 0.511 |
| Employment | 9 (33%) | 5 (36%) | 2 (33%) | 2 (29%) | 1.000 |
| Physical activity | 3 (11%) | 2 (14%) | 0 (0%) | 1 (14%) | 1.000 |
| Provides at least one transportation or mobility service | 12 (44%) | 5 (36%) | 2 (33%) | 5 (71%) | 0.125 |
| Home delivery | 11 (41%) | 4 (29%) | 2 (33%) | 5 (71%) | 0.199 |
| Transportations vouchers or passes | 5 (19%) | 3 (21%) | 1 (17%) | 1 (14%) | 1.000 |
| Mobile markets | 3 (11%) | 2 (14%) | 0 (0%) | 1 (14%) | 1.000 |
| **Overall partnership & Referral Score, median points (IQR)** | 7 (3–13) | 7 (0–13) | 4.5 (3–7) | 11 (6–13) | 0.745 |

**Note:** Fisher's exact tests used for individual practices and “at least one” practice composite scores due to small cell sizes. Overall partnership and referral score uses summed NEFPAT+ Objective 7 scores analyzed with Kruskal-Wallis tests; values shown as median (IQR). Binary coding used for each pantry practice. Composite scores indicate pantries providing “at least one” service per category. No statistically significant differences were observed by pantry size (all p > 0.05).
